# Effect of Steam Deactivation Severity of ZSM-5 Additives on LPG Olefins Production in the FCC Process

**DOI:** 10.3390/molecules22101784

**Published:** 2017-10-21

**Authors:** Andrey A. Gusev, Antonios C. Psarras, Konstantinos S. Triantafyllidis, Angelos A. Lappas, Paul A. Diddams

**Affiliations:** 1 Centre for Research and Technology Hellas, Chemical Process and Energy Resources Institute, 6th km Charilaou-Thermi Road, Thessaloniki 57001, Greece; agusev@cperi.certh.gr (A.A.G.); psarras@cperi.certh.gr (A.C.P.); 2 Aristotle University of Thessaloniki, Department of Chemistry, University Campus, Thessaloniki 54124, Greece; ktrianta@chem.auth.gr; 3 Johnson Matthey Process Technologies, Technology Center, Bourne Blvd, Savannah, GA 31408, USA; Paul.Diddams@matthey.com

**Keywords:** fluid catalytic cracking (FCC), phosphorus containing ZSM-5 additives, hydrothermal deactivation-dealumination, n-alkane cracking, LPG olefins and aromatics, Brønsted/Lewis acidity

## Abstract

ZSM-5-containing catalytic additives are widely used in oil refineries to boost light olefin production and improve gasoline octanes in the Fluid Catalytic Cracking (FCC) process. Under the hydrothermal conditions present in the FCC regenerator (typically >700 °C and >8% steam), FCC catalysts and additives are subject to deactivation. Zeolites (e.g., Rare Earth USY in the base catalyst and ZSM-5 in Olefins boosting additives) are prone to dealumination and partial structural collapse, thereby losing activity, micropore surface area, and undergoing changes in selectivity. Fresh catalyst and additives are added at appropriate respective levels to the FCC unit on a daily basis to maintain overall targeted steady-state (equilibrated) activity and selectivity. To mimic this process under accelerated laboratory conditions, a commercial P/ZSM-5 additive was hydrothermally equilibrated via a steaming process at two temperatures: 788 °C and 815 °C to simulate moderate and more severe equilibration industrial conditions, respectively. *n*-Dodecane was used as probe molecule and feed for micro-activity cracking testing at 560 °C to determine the activity and product selectivity of fresh and equilibrated P-doped ZSM-5 additives. The fresh/calcined P/ZSM-5 additive was very active in C_12_ cracking while steaming limited its activity, i.e., at catalyst-to-feed (C/F) ratio of 1, about 70% and 30% conversion was obtained with the fresh and steamed additives, respectively. A greater activity drop was observed upon increasing the hydrothermal deactivation severity due to gradual decrease of total acidity and microporosity of the additives. However, this change in severity did not result in any selectivity changes for the LPG (liquefied petroleum gas) olefins as the nature (Brønsted-to-Lewis ratio) of the acid/active sites was not significantly altered upon steaming. Steam deactivation of ZSM-5 had also no significant effect on aromatics formation which was enhanced at higher conversion levels. Coke remained low with both fresh and steam-deactivated P/ZSM-5 additives.

## 1. Introduction

Nanoporous materials represent an ideal class of catalytic materials or catalyst supports as they may offer high micro/mesoporous surface areas for efficient dispersion of single-atom active sites or metal nanoparticles, as well as controlled shape-selectivity depending on the type/morphology and size of the nanopores [1,2,3] Zeolites, perhaps the most known and applied microporous materials, are widely used in many catalytic applications. Specifically, ZSM-5 zeolite is used in several important processes in the refining and petrochemical industry due to its high activity and shape selectivity [4,5,6,7,8,9,10,11,12,13,14,15,16,17,18,19]. Fluid catalytic cracking (FCC) is one of the most important processes in a modern refinery as it can provide up to 50% of the total gasoline pool [11,20]. Use of ZSM-5 catalytic additives in the FCC process increases the production of propylene and butylenes that are very important raw materials in the petrochemical industry [11,12,13,14,21,22,23,24,25,26,27,28,29,30]. It must be noted that propylene world demand increases at a rate of 4–5% per year, with 35% of this propylene demand being met by the FCC process [31]. Butylenes are very important for the refineries because they are often used to make high quality gasoline blending components via alkylation or/and ethers (e.g., ethyl-tertiarybutyl ether, ETBE) [11,13,21,25,26]. In addition to optimizing operating variables affecting LPG olefin production in FCC (e.g., high riser outlet temperature and low hydrogen-transfer catalysts) the most important factor to boost light olefin production is use of ZSM-5 additives [8,27,30]. Addition of ZSM-5 offers great flexibility to a refinery since, in a relatively simple and low cost manner, gasoline octane (RONc and MONc) and light olefins yields can be substantially increased [11,12,13,25,26,27,28,29,30]. ZSM-5 functions via two key mechanisms: (1) the cracking of lower octane gasoline normal olefins and paraffins with the simultaneous production of C_3_ and C_4_, LPG, olefins; and (2) via isomerization reactions in which straight chain gasoline components, such as C_7+_ olefins, are converted to more highly branched isomers, which have a greater contribution to octane, especially RONc [10,11,12,21,24,25,26,27,28,29,30,32]. Isomerization reactions are facile on ZSM-5 and persist long after cracking activity is lost following deactivation [13,21,27]. Gasoline range aromatics on a wt % feed basis are virtually unaffected by the presence of ZSM-5, as their concentration increases while the overall gasoline yield decreases. By these two pathways the concentration of low RON compounds is reduced, however at the expense of gasoline yield. 

In the FCC process, the catalyst and additives are subjected to continuous circulation between the riser, where cracking reactions occur, and the regenerator where the coke deposited on the catalyst during cracking is burned off. Coking of ZSM-5 is much lower than coking on the base zeolite USY catalyst because of its lower acid site density (higher silica to alumina ratio, SAR) and smaller micropores network [15,33,34]. Deactivation by coke is called “reversible or temporary deactivation” because the coke is burned off in the regenerator and the deactivation is not permanent. The regeneration environment includes high temperatures and the presence of steam produced from combustion of hydrogen in coke, which together lead to hydrothermal deactivation of the zeolitic components of FCC catalysts and additives. Hydrolysis of the zeolitic framework causes the abstraction of aluminium atoms in a process called dealumination [35,36]. Activity loss is strongly associated with the extent of dealumination of the zeolitic framework and the consequent reduction in Brønsted acidity [14,16,29,34,35,36,37,38,39,40,41,42,43,44,45,46,47,48,49,50,51,52,53,54]. This type of deactivation is irreversible because the catalyst activity is not restored by regeneration and must be compensated for by adjusting the catalyst/additive addition rates. 

In order to evaluate catalytic activity and establish reaction mechanisms in the cracking of petroleum fractions, model compounds such as C_3_–C_12_ paraffins are often tested as feed with ZSM-5, Y and other industrially relevant zeolites [14,15,16,17,18,19,33,46,47,48,49,50,51,52,53,54,55,56,57,58,59,60,61,62,63,64,65,66,67,68,69]. The prevalent model describing alkane cracking over proton zeolites is the Haag-Dessau mechanism which involves the monomolecular protonation of the paraffin on C-H or C-C bonds, by strong Brønsted acid sites, such as those present on ZSM-5 zeolite, forming penta-coordinated carbonium ion intermediates. As it is described by several authors, carbonium ions undergo α-cracking reactions resulting in a smaller paraffin and more stable carbenium ion which can further be desorbed as a smaller alkene [15,16,17,18,56,57,58,59,64,65,66,67,70,71,72,73] . However, the required activation energy for the protonation of a paraffin is very high due to the formation of high-energy transition state [74,75]. Therefore, the stability of carbonium ions is very low, and the rate of reactions proceeding via this route under FCC conditions would be very low and restricted to areas of the FCC unit where thermal cracking reactions are prevalent and free radicals are formed. Owing to the nature of the FCC catalyst formulation and the process conditions, the predominant path in the FCC process is considered to be the bimolecular cracking reactions. It is much more likely that paraffins are activated by forming lower activation energy carbenium ions on Lewis acid sites. Such sites are present on the silica-alumina or alumina “matrix” of the FCC particles or on the zeolite extra-framework aluminum species formed during its hydrothermal dealumination. Once formed, carbenium ions are able to undergo a number of possible reactions, including: β-scission, isomerization, oligomerization, and hydrogen transfer with hydrogen donor molecules such as naphthenes (cycloparaffins) [15,16,17,57,58,59,67,71,72,73].

In this work, the effect of hydrothermal equilibration conditions of ZSM-5 (in the form of FCC additives, where the ZSM-5 crystals are held in silica-alumina matrix) on its activity and selectivity for LPG olefins production was systematically studied by using *n*-dodecane as a probe feed molecule. Moreover, steaming tests of phosphorus-doped H-ZSM-5 zeolite and amorphous silica-alumina, separately, were also performed, in order to elucidate the textural and acidic changes of individual components in ZSM-5 based additive upon steaming deactivation. The structural, porosity, and acidity characteristics of both the fresh and steamed P/ZSM-5 formulations were determined and were correlated to their catalytic performance, leading to the identification of various mechanistic pathways for n-paraffin cracking.

## 2. Results and Discussion

### 2.1. Structural, Porosity, and Acidic Characteristics of ZSM-5 Additives

**Figure 1:**
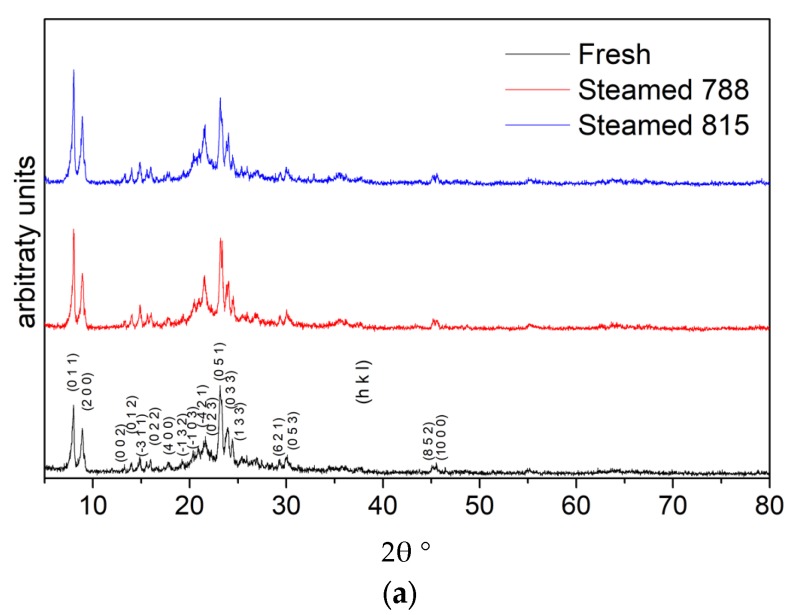

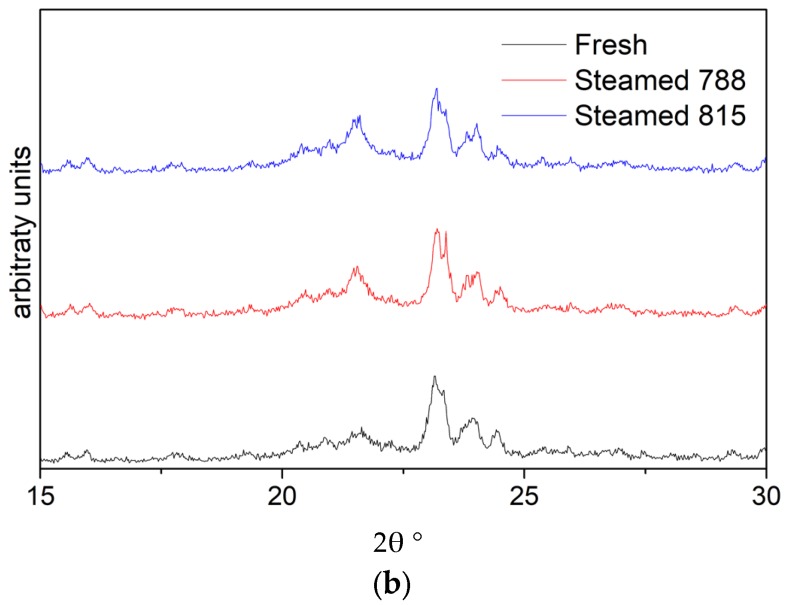
X-ray diffraction (XRD) patterns of the fresh and steamed P/ZSM-5 (at 788 °C and 815 °C for 20 h, 100% steam) additives: (**a**) 5–80° 2θ range; (**b**) and 15–30° 2θ range.

The X-ray patterns of fresh (calcined) and steamed additives, shown in Figure 1, were utilized to verify the characteristic structure of P/ZSM-5 zeolite in the additives and for the assessment of any crystallinity loss upon steaming deactivation. The sum of integrated intensities of the three dominant peaks between 23.1° and 24.3° 2θ were used as an index of the samples’ relative crystallinity, and the respective data are presented in Table 1. As can be seen, the severe steaming treatment of the additives induced a relatively limited decrease of ZSM-5 crystallinity, no higher than ca. 12%. This high resistance to steaming has been also previously shown for pure ZSM-5 zeolites and varying hydrothermal treatment severity [48,49,50,51,69,76]. Steaming of the P-doped pure H-ZSM-5 zeolite of the present study at 770 °C for 7 h under He/steam flow (50% steam) induced also a marginal crystallinity decrease by ca. 14%, as can be seen in Table S1 and Figure S1, in Supplementary Materials. Under the same steaming conditions, the crystallinity reduction of the P/ZSM-5 additive was about 10% (Table S1, Supplementary Materials), similar to those measured under the more severe steaming conditions (Table 1).

The total (BET) surface areas of the ZSM-5 additives are presented in Table 1. The total surface area did not change significantly (within experimental error) upon steam equilibration at 788 °C. Moreover, when the steaming treatment became more severe, the total surface area decreased. Although, these changes are relatively small and close to the experimental error of the method, substantial changes occur within the micropore and meso/macropore structure. A 34% decrease of micropore area, calculated by t-plot method, was measured after steaming at 788 °C for 20 h, which increased to 39% at 815 °C steaming. A similar trend was observed for the micropore volume. On the other hand, the meso/macropore (including external) surface areas for both steamed samples were more than doubled, compared to that of the fresh ZSM-5 additive. This clearly shows that there is a process of meso/macropores formation in P/ZSM-5 additives during the steaming procedure. Similar results were also reported by other groups for P-doped ZSM-5 additives [39,44].

**Figure 2:**
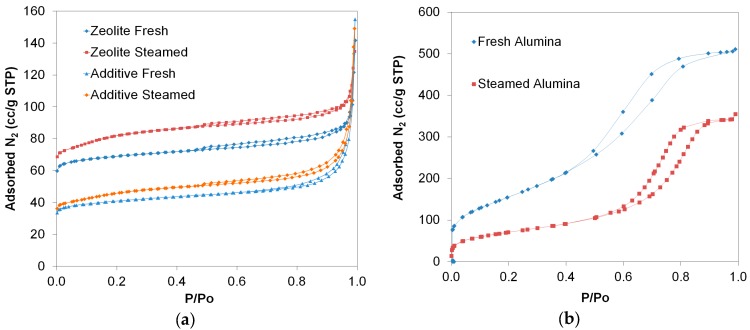
N_2_ adsorption-desorption isotherms for: (**a**) P/H-ZSM-5 zeolites (fresh and steamed) and P/ZSM-5 additives (fresh and steamed); (**b**) silica-alumina (fresh and steamed); steaming conditions 770 °C, 7 h, He/steam flow (50% steam).

In order to further study the effect of steaming on the textural properties of the P/ZSM-5 additive, a P-doped H-ZSM-5 pure zeolite and a silica-alumina sample were separately steamed at relatively severe conditions (770 °C, 7 h, He/steam flow, 50% steam), as described above. The obtained N2 isotherms are shown in Figure 2 and the calculated porosity data are given in Table 2; the BJH pore size distribution curves are also provided in Figure S3 (in Supplementary Materials). The P/ZSM-5 additive was also subjected to the above steaming treatment for comparison. As can be seen in Figure 2, the N_2_ adsorption isotherm of the fresh-calcined P-doped ZSM-5 zeolite is typical for microporous materials (type I(a) isotherm according to the new IUPAC classification [77] exhibiting a steep increase of N_2_ sorption at very low partial pressures (i.e., P/Po ≤ 0.01) due to micropores filling. However, instead of the characteristic plateau at higher P/Po, a progressive small increase of N_2_ sorption occurred up to ca. P/Po = 0.95 after which N_2_ increased sharply again (typical for type II isotherms). These observations can be attributed to defects of the zeolitic crystals, as well as to the presence of loosely aggregated small nanocrystals, both providing voids in the range of meso- and macropores as well as high external surface area. In fact, in most cases, both the laboratory synthesized and industrially prepared zeolites exhibit such type of “non-ideal” isotherms and microporous structures. Based on the above described morphology of the zeolite crystals, the observed hysteresis loop can be classified as being of H4 type attributed to aggregated zeolitic crystals or even intracrystal mesopores [77]. The shapes of adsorption isotherm and hysteresis loop of the steamed P-doped ZSM-5 zeolite are similar to those of the fresh zeolite, with the exception of an additional step of slightly enhanced N_2_ sorption observed at P/Po = 0.01–0.2, which can be attributed to super-micropores or small mesopores (of ca. ≤2.5 nm). With regard to the adsorption isotherm of the P/ZSM-5 additive, it is also similar to that of the pure P/ZSM-5 zeolite except that in the case of the additive, there is a stronger contribution of type II isotherm due to the presence of the meso/macroporous silica-alumina matrix. Accordingly, the hysteresis loop is a mixture of type H4 and H3, the latter being representative of not completely filled macropores or non-rigid aggregates of plate-like particles [77]. 

By comparing the data of Table 1 and Table 2, it can be seen that the observed changes and trends in porosity were similar when the P/ZSM-5 additive was steamed at 788 °C for 24 h under 100% steam and at 770 °C for 7 h under He/steam flow, 50% steam, with a relatively stronger effect on microporosity at the more intense conditions. More specifically, by steaming the additive at 770 °C the total surface area changed from 159 m^2^/g to 167 m^2^/g (to 157 m^2^/g upon steaming at 788 °C), the micropore area decreased from 122 m^2^/g to 92 m^2^/g (to 80 m^2^/g at 788 °C) and the meso/macropore area increased from 37 m^2^/g to 75 m^2^/g (to 77 m^2^/g at 788 °C). These data verified the trend discussed above, of the initial marginal change of total surface area of the additive, at steaming temperature below ca. 790 °C, due to the substantial increase of the meso/macropore area which compensated the decrease of micropore area. This trend was observed when the pure P/H-ZSM-5 zeolite was steamed at 770 °C, 7 h, He/steam flow, 50% steam, i.e., the total surface area was increased (270 to 304 m^2^/g) since the decrease of micropore area (225 m^2^/g to 201 m^2^/g) was compensated by the substantial increase of meso/macropore area (45 to 103 m^2^/g), as shown in Table 2. Previously, it was reported that the hydrothermal treatment, at relatively severe conditions as the above, of phosphorus free ZSM-5 zeolites reduced the total surface area [49,50,52,53,69,76] due to reduction of the micropore area. With regard to the P-containing ZSM-5’s, it appeared that relatively moderate P loadings (i.e., 2 wt %) stabilized the microporous structure, leading to minimum decrease or even increase of micropore volume upon steaming, depending of the Si/Al of ZSM-5 [53]. It is also proposed that P-containing zeolites and catalysts may have their zeolitic micropores blocked partially by phosphorus species, while the steaming procedure induced the removal of phosphorus species from micropores [44,69]. As far as the amorphous silica-alumina is concerned, it can be seen that steaming reduced its total surface area which was solely attributed to meso/macropore area, both in the fresh and steamed sample (Table 2 and Figure 2b). It can thus be suggested that the changes in porosity of the P/ZSM-5 additive upon steaming at FCC relevant conditions, are mainly attributed to the changes of the zeolitic crystals, i.e., the decrease of microporosity, and the formation of secondary meso/macropore network. Still, however, there are cases of silica-alumina matrices which upon steaming may exhibit enhanced surface area compared to their parent calcined form. 

**Figure 3:**
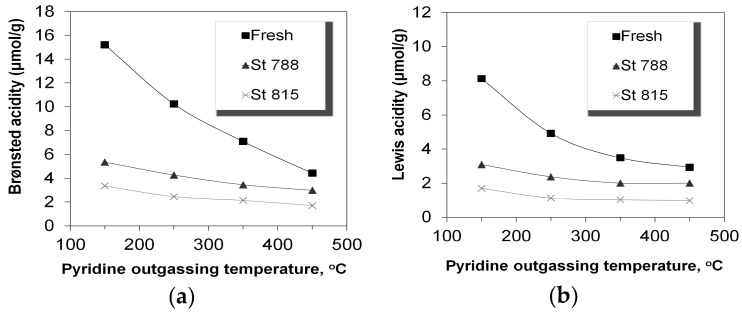
Acidity of the P/ZSM-5 additives: (**a**) Brønsted and (**b**) Lewis determined by FTIR-pyridine measurements at increasing pyridine equilibration temperature.

With regard to acidity, the pyridine sorbed at 150 °C represents the total amount of acid sites (Table 1). The curves in Figure 3 show the relative adsorption strength of the Brønsted and Lewis acid sites, as expressed by the amount of pyridine remained sorbed on the ZSM-5 additives at increasing outgassing/equilibration temperature in the FTIR vacuum cell. Steaming of the P/ZSM-5 additives resulted in a significant decrease of both Brønsted and Lewis acid sites, which became stronger at higher steaming severity. The reduction of Brønsted acid sites was more pronounced and was mainly related to dealumination of the zeolitic framework, as has been previously shown [40,51,53,69,76,78]. When the pure P/ZSM-5 zeolite was steamed, its Brønsted acidity was substantially reduced and concurrently its Lewis acidity slightly increased due to the framework aluminium species ejection, as described above (Table 2). As discussed in the introduction, ZSM-5 additives also possess Lewis acidity in addition to Brønsted acidity, even in the “fresh” calcined form. These Lewis sites are mainly due to the silica–alumina matrix, but may also be due to the presence of zeolitic extra-framework alumina phases that are generated during the initial calcination step for organic template combustion. As can be seen from the data in Table 1, the Brønsted to Lewis (B/L) acid sites ratio was ~2 for the fresh P/ZSM-5 additive and remained at this ratio for the steamed additives. Furthermore, the steeper decrease of sorbed pyridine for the fresh P/ZSM-5 additive with increasing outgassing temperature, compared to that for the steamed samples, indicates that the latter have higher portion of strong acid sites (Figure 3). In the case of Brønsted acidity of the fresh P/ZSM-5 additive, the contributing sites can be those originating from the aluminium atoms in the zeolitic framework (relatively strong acid sites) and the SiAlSi-OH of the amorphous silica-alumina matrix (weak acid sites). The presence of the latter type of acid sites was responsible for the relatively low acid strength of the ZSM-5 additives indicated by the FTIR pyridine experiments, in contrast to the Brønsted acidity of pure ZSM-5 zeolites which usually exhibit high adsorption strength [33]. In addition, it has been reported that phosphorous blocks/neutralize the Brønsted acid sites in the fresh-calcined ZSM-5 zeolite but acts as “protecting” agent of framework aluminium during steaming, resulting in higher acidity of the steamed P-doped ZSM-5 compared to the steamed analogue without P addition [52,53,69]. The interaction of extra-framework alumina phases, formed upon steam dealumination at relatively mild conditions, with the zeolitic framework Brønsted sites may also induce higher acidity strength and catalytic activity, as has been also previously suggested [65,78] All the above can be considered in order to rationalize the increased strength of the Brønsted acid sites in the steamed P-doped ZSM-5 additives. With regard to the Lewis acidity, its increased strength in the steamed ZSM-5 additives may be related to the more condensed alumina or silica-alumina phases formed under these high temperature/steam conditions. 

### 2.2. Cracking of n-Dodecane (n-C_12_)

**Figure 4:**
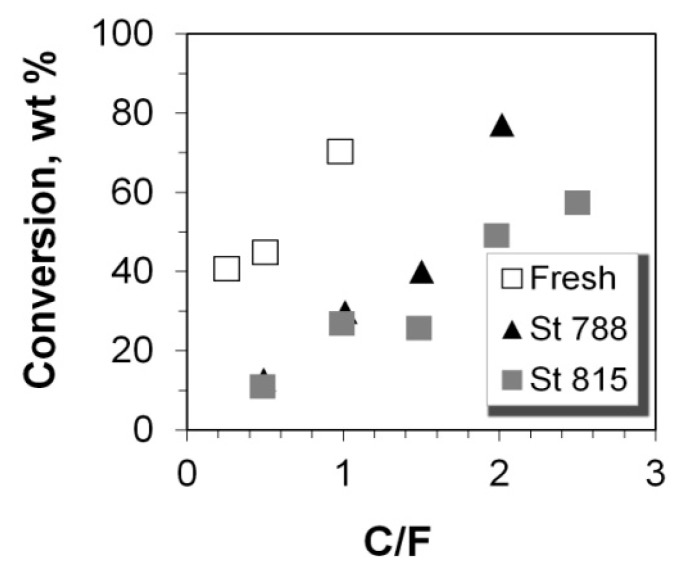
Conversion (wt %) of n-C_12_ versus catalyst-to-feed (C/F) ratio for the fresh and steamed P/ZSM-5 additives.

**Figure 5:**
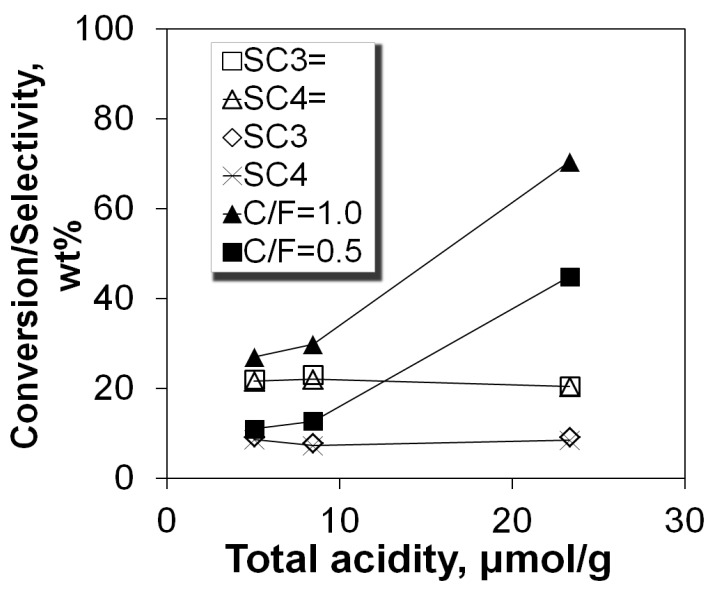
Correlation of n-C_12_ conversion (C/F 0.5 and C/F 1.0) and product selectivities (propylene, total butylenes, propane, and total butanes) with the total acidity of the P/ZSM-5 additives (LPG selectivity values refer to ~47% constant conversion).

The cracking activity of the P/ZSM-5 additives was evaluated in the SR-SCT-MAT unit (described in experimental section) by determining the percent conversion of the n-C_12_ feed. In all experiments, the mass balance was higher than 95%. The results of conversion versus the catalyst-to-feed mass ratio for the fresh and steam equilibrated additives are depicted in Figure 4. The fresh additive was significantly more active compared to the two steamed additives, due to the higher number of acid sites of the former, and possibly due to its higher micropore surface area/volume that offered a favorable confined space for increased n-paraffin conversion rates [79]. It appears that the higher acid strength of the steamed P/ZSM-5 additives, as indicated by the FTIR-pyridine experiments, was not sufficient to compensate for the 3-times lower number of their acid sites compared to those of the fresh additive. As it was described above, the activation energy for protolytic cracking is energetically demanding due to the high-energy transition state [74,75]. Therefore, the cracking rate of monomolecular reaction is very sensitive to the strength of Brønsted sites [80]. If this mechanism was prevalent then the differences in activity between the samples would not be significant. It can thus be suggested that the prevalent cracking mechanism was the classical bimolecular β-scission. Furthermore, the two steamed P/ZSM-5 additives exhibited similar conversion activity, as expected, due to their similar acidic and porosity characteristics. The correlation between the total acidity of the P/ZSM-5 additives and their conversion activity, at the two C/F tested, can be clearly seen in Figure 5.

### 2.3. Selectivity to Dry Gases

**Figure 6:**
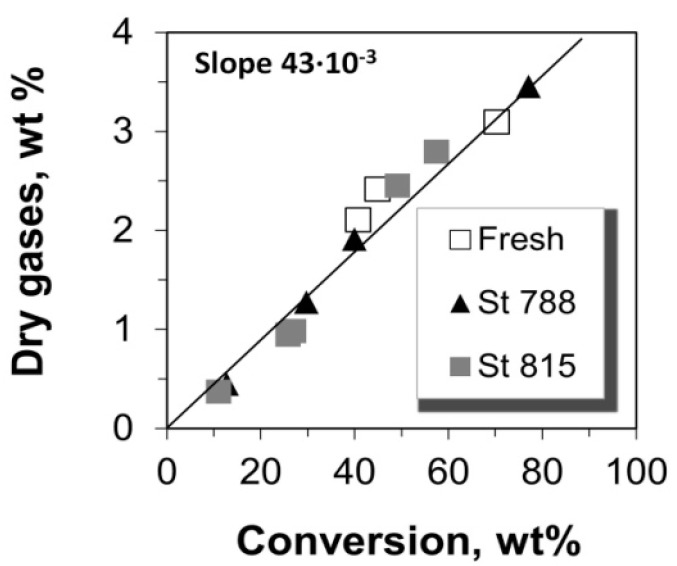
Yield of total dry gases versus the conversion of n-C_12_.

**Figure 7:**
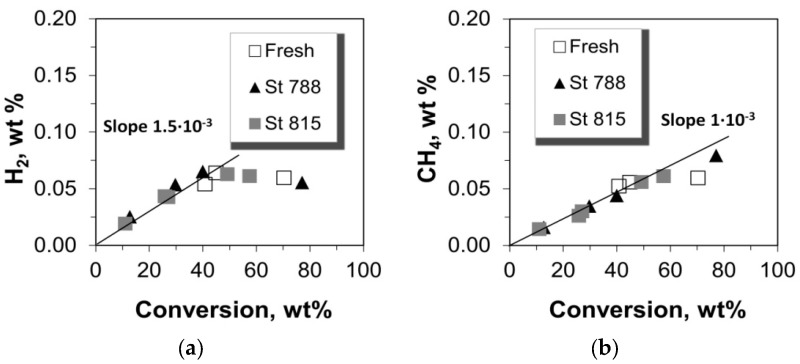
Production yields of (**a**) hydrogen and (**b**) methane versus the conversion of n-C_12_.

**Figure 8:**
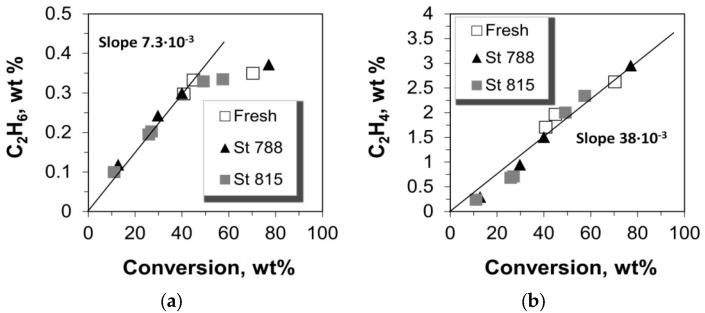
Production yields of (**a**) ethane and (**b**) ethylene versus the conversion of n-C_12_.

The yield of total dry gases, as well as the yields of the individual gases i.e., hydrogen, methane, ethane, and ethylene versus conversion are shown in Figure 6. The yield of total dry gases increases monotonically with the conversion of n-C_12_, however, this is attributed mainly to ethylene which has the highest concentration in the total dry gases and exhibits a linear increase with conversion. Hydrogen, methane, and ethane are produced at much lower level and they show a progressive increase with conversion values of up to ca. 40%, after which they remain almost constant (Figure 7 and Figure 8). Such a cracking behavior indicates that the Haag-Dessau mechanism [15,16,17,18,56,57,58,59,64,65,66,67,70,71,72,73] was not the predominant one despite the feed being a saturated molecule, at least in the case of n-C_12_ cracking with the fresh and steamed P/ZSM-5 commercial additives of this study. As discussed above, the Haag-Dessau mechanism of protolytic alkane cracking at about 530 °C on acidic catalysts such as ZSM-5 zeolite, involves the protonation of an alkane to form carbonium ion intermediates which then crack to produce smaller alkanes (methane and ethane) and hydrogen, and carbenium ions, which give back protons to the catalyst to form alkenes. It is more likely that cracking of n-C_12_ alkane proceeds via hydride abstraction on Lewis acid sites and formation of carbenium ion intermediates, being propagated via the classical bimolecular cracking pathway, as explained in more detail in Section 2.7 below. Protolytic cracking at Brønsted acid sites becomes kinetically significant as the concentration of alkenes increases. Based on the above, the production of small amounts of hydrogen, methane, and ethane can be mainly attributed to thermal cracking reactions where high energy radicals are present. The activation energy of such reactions is very high and therefore the kinetics are quite slow.

The amount of ethylene produced is far higher than all the other dry gas products (Figure 7 and Figure 8). Moreover, it is much more ‘sensitive’ to catalyst mass and to the increase of conversion via increased catalyst-to-feed (C/F) ratio. The slopes of the lines “Yield vs. Conversion” (in Figure 7 and Figure 8) for up to 40% conversion were estimated as can be seen in Figure 7 and Figure 8; only in the case of ethylene the whole range of conversion was taken into account due to its linearity over the whole range of conversion values. The values of these slopes show that the selectivity of ethylene is much higher than that of the other thermal cracking products. In the classical carbenium ion cracking mechanism on zeolitic Brønsted acid sites, ethylene production can be only justified via primary carbenium ion formation which is not favorable energetically. Such a high ethylene production has been also previously reported in vacuum gas-oil (VGO) cracking tests with fresh and dealuminated/steamed ZSM-5 zeolites used as FCC catalyst additives, where the contribution of the mechanism of protolytic cracking of alkanes was also considered as minimum [10]. Thus, the increased selectivity and production of ethylene may be associated with more energetically favorable intermediates via the radical formation mechanism in thermal cracking, without excluding the contribution of catalytic cracking reactions on ZSM-5. 

### 2.4. Selectivity of LPG Products

**Figure 9:**
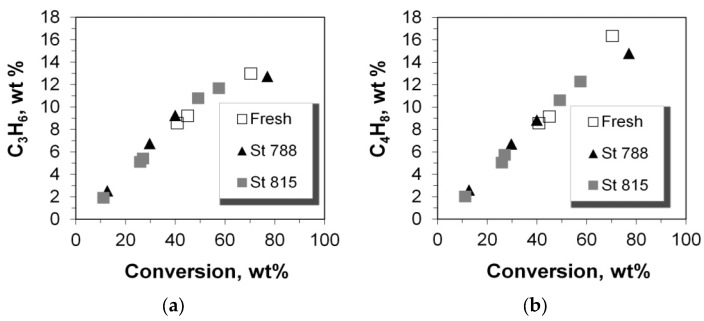
Production yields of (**a**) propylene and (**b**) total butylenes versus the conversion of n-C_12_.

**Figure 10:**
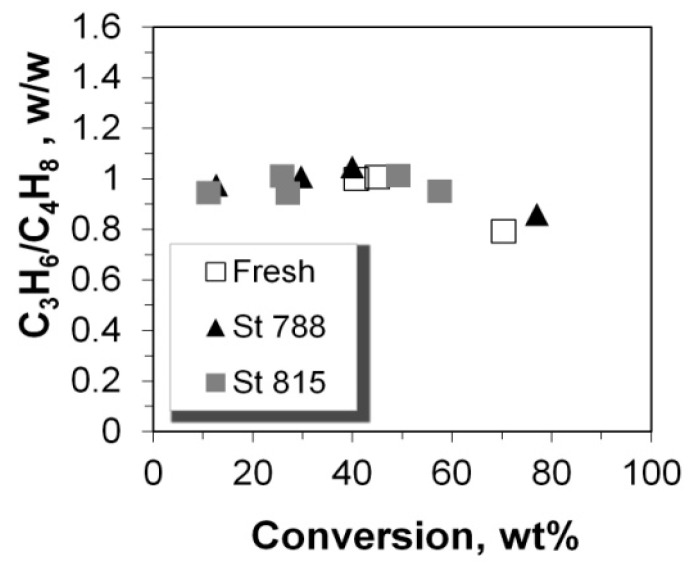
Mass ratio of propylene over butylenes versus the conversion of n-C_12_.

The yields of propylene and total butylenes versus conversion are shown in Figure 9 and Figure 10. Both increased monotonically with conversion, except in the case of propylene, at conversion values higher than ca. 60%, where this linear increase was interrupted, possibly due to their transformation to aromatics, as is discussed below. This change in selectivity/yield affected the mass ratio of total butylenes to propylene, which is almost constant for conversion up to ca. 60%, but increases at higher values (Figure 10). Interestingly, all (fresh and steamed) P/ZSM-5 additives obeyed the same average correlation line. It can thus be suggested that the active acid sites on both the fresh and steamed additives that participate in the formation of propylene and butylenes are of the same type, thus providing similar product selectivity. This is further supported by the similar Brønsted to Lewis (B/L) ratios measured for the fresh and steamed additives (Table 1). Accordingly, from the data in Figure 5, it can clearly be seen that despite the increase of conversion with increasing total acidity, the selectivity to various LPG alkenes and alkanes remains almost constant at constant conversion.

**Figure 11:**
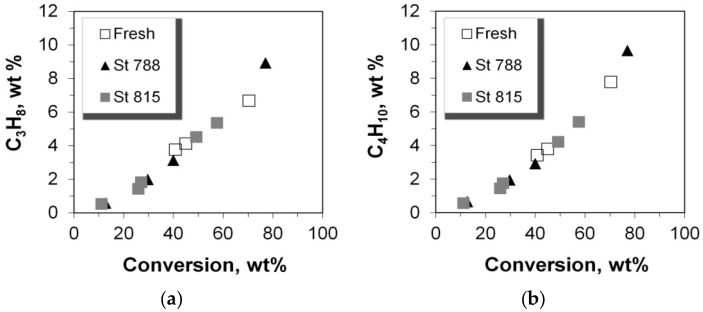
Product yields of (**a**) propane and (**b**) butanes versus the conversion of n-C_12_.

**Figure 12:**
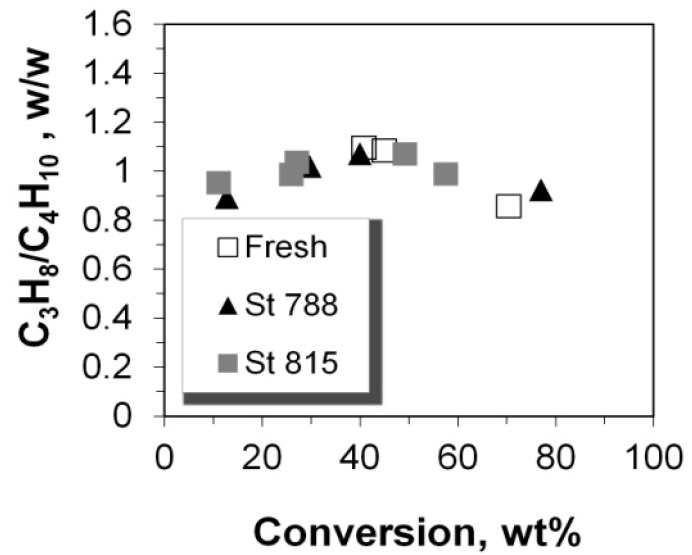
Mass ratio of propane over total butanes versus the conversion of n-C_12_.

The yield of propane increased linearly with conversion, except with a small positive deviation at very high conversion of ca. 70% (Figure 11) which could be associated with the reactivity of Lewis acid sites that may favor the production of smaller alkanes in n-paraffin cracking. The positive effect of high conversion on small alkane yields, was also identified in the case of butanes for all the P/ZSM-5 additives tested, and becomes slightly more pronounced at 40% conversion and upwards (Figure 11). Thus, at low conversion of up to ca. 40%, the mass ratio of propane to butanes increases slightly from 0.9 to 1.1 but at conversion higher than 40%, this ratio decreases due to the enhanced production of butanes (Figure 12). 

### 2.5. C_2_–C_5_ Olefinicities

**Figure 13:**
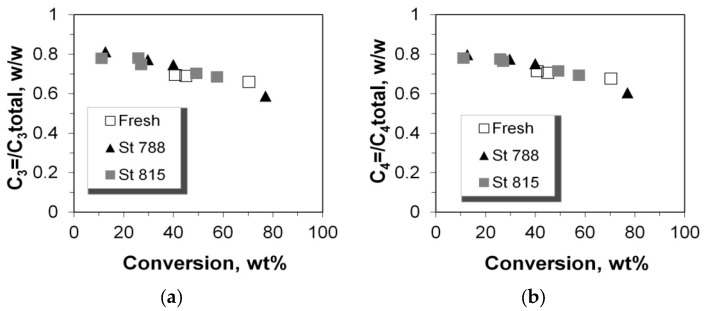
Olefinicity ratios for LPG range products: (**a**) C_3_; (**b**) C_4_.

**Figure 14:**
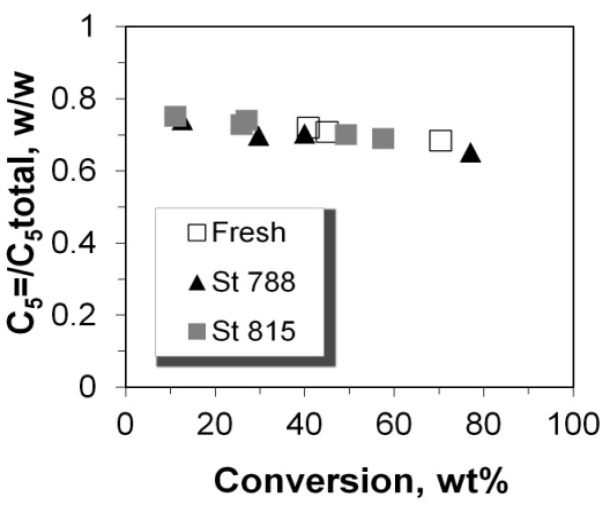
Olefinicity ratio for C_5_ products.

**Figure 15:**
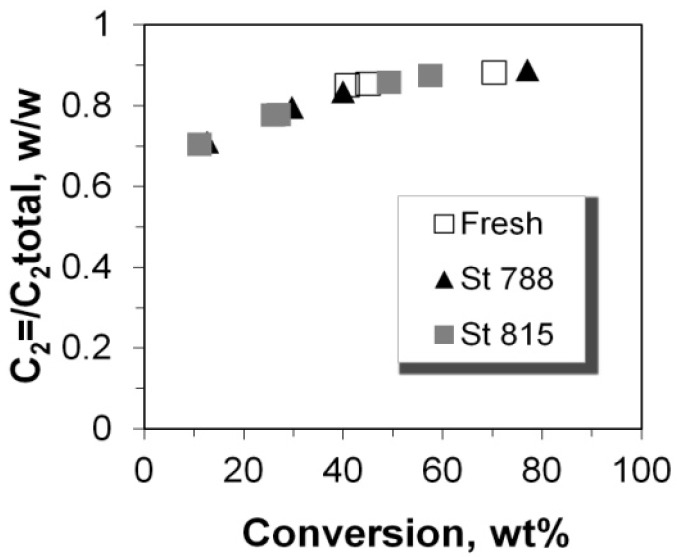
Olefinicity ratio for C_2_ products.

The olefinicity of the products is an important parameter related to the economics of the FCC process and is defined as the mass ratio of olefins (alkenes) over the sum of paraffins (alkanes) plus olefins with the same carbon number. The general trend for C_3_, C_4_, and C_5_ olefinicity is to decrease vs. C/F ratio (and conversion), as these alkenes are highly reactive mainly towards aromatics, coke, as well as small alkanes at relatively high conversion values (Figure 13 and Figure 14). On the other hand, ethylene’s olefinicity increases with catalyst mass and conversion degree (Figure 15) perhaps due to a continuously increasing contribution from thermal and catalytic cracking as C/F increases. Another important observation is that for all products, the olefinicity data for all the P/ZSM-5 additives, obey the same correlation curves, thus supporting again the argument that the nature and balance (Brønsted to Lewis sites) of the acid active sites are the same in the fresh and the steamed additives, irrespective of steaming severity. Only their number is different, and this is reflected mainly on the conversion activity.

### 2.6. Production of Coke and Aromatics

**Figure 16:**
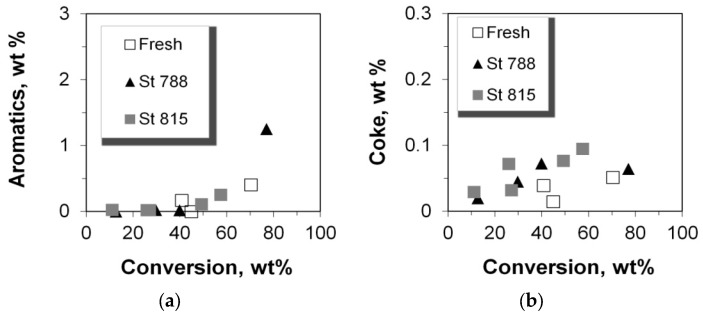
Product yields of: (**a**) aromatics and (**b**) coke versus the conversion of n-C_12_.

As suggested above, olefins are consumed towards the formation of aromatics. From the data in Figure 16a, it can be clearly seen that aromatics started to form at 40% conversion and higher, and increased exponentially at very high conversion degrees, even with the steamed P/ZSM-5 additives, in accordance with the lower production of propylene at such high conversion. Furthermore, the increased selectivity toward butanes at conversion levels of 40% and higher maybe also associated with the commencing of C_3_ aromatization reactions. Interestingly, aromatics appear to be somehow stable to oligo/polymerization, since coke is not significantly affected/increased with conversion (Figure 16b). This is in line with previous works which showed the relatively low coke selectivity of ZSM-5 compared to other zeolites [15].

### 2.7. Cracking Mechanism

**Figure 17:**
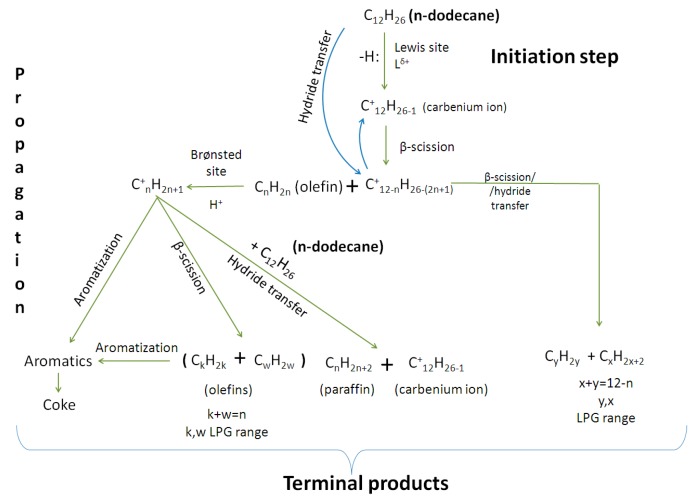
Reactions network for n-C_12_ cracking over P/ZSM-5 additives (including SiO_2_/Al_2_O_3_ matrix).

Based on the above observations, a reaction pathway scheme for the cracking of n-C_12_ on P/ZSM-5 additives can be proposed (Figure 17). The initiation step involves a Lewis acid site (either from the silica-alumina matrix or from the extra-framework Al species of the ZSM-5 zeolite) in order to form a carbenium C_12_^+^ ion as intermediate species. The reaction then propagates via β-scission of the carbenium ion to produce a smaller alkene and a smaller carbenium ion. The formed alkene can either desorb in the product stream or can be protonated on a Brønsted acid site to form a new, smaller (than the n-C_12_^+^) carbenium ion. A third pathway mainly for C2= and C3= alkenes is their aromatization (via oligomerization/cyclization and dehydrogenation) on the Brønsted acid sites. The alternative main reaction pathway, instead of β-scission, is the hydride transfer (bimolecular route) from n-C_12_ (feed) or any formed smaller alkane to the sorbed carbenium ions (n-C_12_^+^ and smaller), which leads to the formation of new smaller alkanes (as well as n-C_12_) and a new carbenium ions. All the above pathways may propagate to the smallest possible (energetically and kinetically favored) alkanes and alkenes, and ore aromatics and coke. 

## 3. Experimental

### 3.1. Catalysts

In the current study, three components were used. A commercially available ZSM-5 additive was supplied by Johnson Matthey. This additive contains silica-alumina as “matrix” and ZSM-5 zeolite (SAR = 30). The additive was in micro-spheres form, prepared by classical industrial spay-drying method when phosphorus was also added via impregnation by homogenization of the feed slurry. For this reason, this additive is named in this work as P/ZSM-5 additive. This “as received” additive was calcined in a muffle furnace in air for 1 h at 732 °C, to ensure complete organic template removal. This calcined sample is indicated as “fresh” in the following characterizations and catalytic experiments. The other material was H-ZSM-5 zeolites with SAR 30 doped with phosphorus (8 wt % P_2_O_5_) and it was supplied by Johnson Matthey. Moreover, the amorphous silica-alumina supplied by Grace Davison (DAVICAT SIAL 3113) was also used for steaming studies, as described below.

The as-received additive was hydrothermally treated (steamed) at two temperatures, i.e., 788 °C and 815 °C for 20 h under 100% steam which acted as the fluidization gas. The experiments were carried out in a bench scale steaming unit consisting of a quartz fluid bed reactor heated in a 3-zone furnace. The capacity of the reactor for the steaming procedure was 200 g catalyst per batch. Temperature control was achieved by means of an internal thermocouple in the catalyst bed. After calcination or steaming, the fresh and steam equilibrated samples were sieved and only the particles in the 45–106 μm range were used for catalytic experiments.

Steaming deactivation tests of individual components, i.e., P/H-ZSM-5 and amorphous SiO_2_/Al_2_O_3_ were also conducted in a smaller bench-scale fluid-bed unit, with 3.5 cm o.d. quartz reactor containing a fritted disc. In a typical test, 6 g of the catalyst was treated with steam at 770 °C for 7 h. A three-zone radiant heater furnace was used to heat up the reactor, while the actual steaming temperature was measured by a thermocouple placed inside the catalytic bed. Steam was introduced in the feed mixture by the aid of He flow (150 cc/min) via a saturation water bath heated at 83 °C (steam partial pressure was ~0.5 bar). These steaming conditions were not identical to those of the P/ZSM-5 additive but were sufficient for studying the structural and textural changes of pure ZSM-5 zeolite that occurred under the relatively severe steaming in the FCC regenerator. 

### 3.2. Characterization

Fresh and steam equilibrated ZSM-5 samples were characterized regarding their surface area, Brønsted and Lewis acidity, and crystallinity. Specific surface area of the catalysts was determined by nitrogen physisorption at −196 °C, using an Autosorb-1 Quantachrome porosimeter (Quantachrome instruments, Boynton Beach, FL, USA). The catalyst samples were outgassed at 300 °C for 16 h under 6.6 × 10^−9^ mbar vacuum prior to N_2_ sorption. The BET multi-point method was used to determine the total surface area while the micropore area and volume were estimated by the t-plot method. The crystalline structure of the catalysts was determined by powder X-ray diffraction (XRD) on a Siemens D 500 diffractometer (Siemens, Karlsruhe, Germany) operated at 40 kV and 30 mA with Cu Kα radiation (λ = 0.154 nm). Diffraction patterns were recorded in the 5–80 2θ range and at a scanning rate of 0.01 s^−1^. The determination of the relative crystallinity of ZSM-5 samples was based on the standard procedure ASTM D5758, as described in Supplementary Materials. 

Acidity was quantified by FTIR coupled with in situ pyridine adsorption. IR spectra were collected using a Nicolet 5700 FTIR spectrometer (resolution 4 cm^−1^) (Thermo Electron scientific instruments corp., Madison, WI, USA) by means of OMNIC software (Prof. 7.0, Thermo Electron scientific instruments corp., Madison, WI, USA). Samples were finely ground in a mortar and pressed in self-supporting wafers (15 mg/cm^2^). The wafers were placed in a custom made stainless steel vacuum cell with CaF_2_ windows. High vacuum is reached by means of a turbomolecular pump and diaphragm pump placed in line. The infrared cell was equipped with a holder surrounded by a heating wire for heating the samples and connected to the vacuum line, which was also heated in order to avoid pyridine condensation or its adsorption on the walls. Before IR analysis, all samples were pretreated at 450 °C under high vacuum (10^−6^ mbar) for 1 h in order to desorb physisorbed species (activation step). Spectra were collected at 150 °C in order to eliminate the possibility of pyridine condensation. Initially, a reference spectrum of the activated sample prior to introduction of pyridine was collected. Then, pyridine was introduced in pulses for 1 h at 1 mbar to allow adsorption onto the acid sites of the catalysts. Pyridine adsorption/desorption was monitored step-wise by evacuating the sample for 30 min at 150, 250, 350, and 450 °C and then cooling back down to 150 °C after each step to record the corresponding spectrum. Stronger acid sites require higher temperatures for the pyridine to be desorbed. The bands at 1545 cm^−1^ (pyridinium ions) and 1450 cm^−1^ (coordinated pyridine) were used to identify and quantify the Brønsted and Lewis acid sites, respectively, by adopting the molar extinction coefficients provided by Emeis [81].

### 3.3. Catalytic Cracking Experiments

A series of cracking experiments were conducted using both the fresh and hydrothermally equilibrated P/ZSM-5 additives in a Single Receiver-Short Contact Time-Microactivity Test (SR-SCT-MAT) unit [82]. The “MAT” unit was operated typically at 560 °C with a feed injection time of 12 s and consisted of the following main modules: the feeding system, the reactor, and the product collection system. The feed was preheated at 30 °C and was injected into a metallic reactor with a special motor pump through the oil capillary for a constant time of 12 s. The metallic reactor was heated by a three-zone furnace and consisted of an annular bed where varying quantities of catalyst were diluted with inert glass beads, so that a constant catalytic bed volume was achieved. Catalyst-to-feed ratios were adjusted by varying the quantities of catalyst (between 0.25–2.5 g) and inert beads used in a fixed bed volume (10 mL) for constant amount of oil injected (1.8 g). Following the feed injection, N_2_ was passed into the reactor in order to flush the products. A specially designed quartz single receiver was connected to the reactor outlet. The vapor and liquid products of cracking reaction were both collected in that single receiver which was placed into a liquid bath (bath temperature 18 °C).

*n*-Dodecane was used as the feed probe molecule for the cracking activity and LPG olefins selectivity study. The reaction temperature, the feed mass, and the injection time of the feed were kept constant and only the catalyst mass was varied. Thus, the catalytic activity and selectivity were presented as a function of catalyst-to-feed (C/F) ratio.

### 3.4. Product Analysis

The gaseous products were analyzed using a specially designed GC called Refinery Gas Analyzer (HP-5890) (Agilent Technologies, Santa Clara, CA, USA). This GC was equipped with four columns and two detectors (TCD and FID). Analysis of the condensed phase (liquid product) and determination of the individual hydrocarbon concentration was conducted by Detail Hydrocarbon Analyser (DHA) Agilent 6890 (Agilent Technologies, Santa Clara, CA, USA). The various hydrocarbon products were lumped as dry gases (H_2_, methane, ethane, ethylene), LPG (liquified petroleum gases, including propane, propylene, butanes and butylenes), total C5s (including C5s measured both in gases and liquid), and aromatics (total C_6_–C_12_ aromatics in gasoline boiling range). The amount of coke, deposited on the catalyst, was measured by an Elemental Analyzer (LECO CS-400 model) (LECO corp., Saint Joseph, MI, USA). 

## 4. Conclusions

The hydrothermal treatment of P/ZSM-5 additives, at relatively intense conditions that simulate the steam deactivation/equilibration in the FCC regenerator, did not affect strongly their crystallinity in contrast to their acidity and porosity characteristics which were substantially altered mainly due to the changes in the zeolite. Specifically, steaming increased the macro/mesopore area of the additives while decreasing their micropore area, in accordance with the behavior observed when a pure P/ZSM-5 zeolite was steam treated. Both Brønsted and Lewis acid sites were reduced but their ratio was almost constant, i.e., ~2 for the fresh and steam equilibrated samples. The fresh P/ZSM-5 additive was very active in C_12_ cracking but steaming resulted in substantial reduction of activity, which can be associated to the decrease of total acidity and microporosity. On the other hand, the approximately constant B/L ratio was consistent with the observation that the selectivity towards LPG alkanes and alkenes (up to ~60% conversion) was nearly constant and independent of ZSM-5 acidity. Thus, the yield of all LPGs increased with conversion and catalyst/feed (C/F) ratio following the same trend for both the fresh and the steamed P/ZSM-5 additives. The yields of propylene and butylenes at conversion levels up to ca. 60% were identical, while at higher conversions, reduced yields of propylene were observed due to its enhanced aromatization on Brønsted acid sites and/or due to the presence of Lewis acid sites taking part in the production of light alkanes. The olefinicities of C_3_–C_5_ products were not affected by steam deactivation of ZSM-5 and were decreased with conversion and C/F ratio indicating olefin consumption and simultaneous paraffin production. The formation of aromatics was also not affected by steaming equilibration of ZSM-5, being significantly enhanced at high conversion degrees (due to high C/F ratio). Coke formation remained at low levels and did not increase with C/F ratio.

## Figures and Tables

**Table 1:** Porosity, crystallinity, and acidity characteristics of the fresh and steamed P/ZSM-5 additives.

Catalyst & Treatment	Total (BET) Surface Area (SA) (m^2^/g)	Micro-Pore SA ^(1)^ (m^2^/g)	Macro/Meso-Pore & External SA ^(2)^ (m^2^/g)	Micropore Volume ^(1)^ (cc/g)	Relative Crystallinity (%)	Brønsted Acidity (μmol Pyridine/g) at 150 °C	Lewis Acidity (μmol Pyridine/g) at 150 °C
“Fresh”(Calcined at 732 °C /1 h)	159	122	37	0.047	100	15	8
Steamed (788 °C/20 h)	157	80	77	0.032	98	5	3
Steamed (815 °C/20 h)	150	74	76	0.029	92	3	1.7

^(1)^ t-plot method; ^(2)^ difference between total and micropore area.

**Table 2:** Porosity characteristics of the fresh and steamed ^(1)^, ZSM-5 zeolites, ZSM-5 additives, and amorphous silica-alumina.

Catalyst	Total (BET) Surface Area (m^2^/g)	Micropore Surface Area ^(2)^ (m^2^/g)	Micropore Volume ^(2)^ (cc/g)	Meso/Macropore & External Surface Area ^(3)^ (m^2^/g)	Brønsted Acidity (μmol Pyridine/g) at 150 °C	Lewis Acidity (μmol Pyridine/g) at 150 °C
P/H-ZSM-5 zeolite	270	225	0.087	45	67	4
P/H-ZSM-5 zeolite steamed	304	201	0.082	103	25	5
SiO_2_-Al_2_O_3_	579	-	-	579	N/A	N/A
SiO_2_-Al_2_O_3_ steamed	260	-	-	260	N/A	N/A
P/ZSM-5 additive	159	122	0.047	36	N/A	N/A
P/ZSM-5 additive steamed	167	92	0.038	75	N/A	N/A

^(1)^ Steaming conditions: 770 °C, 7 h, He/steam flow (50% steam); ^(2)^ t-plot method; ^(3)^ difference between total and micropore area.

## Supplementary Materials

The supplementary materials are available online.
