# Supplementary material for: Effect of Steam Deactivation Severity of ZSM-5 Additives on LPG Olefins Production in the FCC Process
Source: Molecules. 2017 Oct 21;22(10):1784. doi: 10.3390/molecules22101784 (PMC6151522; doi:10.3390/molecules22101784)
Supplement: Supplementary file 1 [file molecules-22-01784-s001.pdf]

Effect of Steam Deactivation Severity of ZSM-5 additives on LPG Olefins Production in the FCC process

Andrey A. Gusev [1,2], Antonios C. Psarras [1], Konstantinos S. Triantafyllidis [2], Angelos A. Lappas [1,*], Paul A. Diddams [3]

[1] Centre for Research and Technology Hellas, Chemical Process and Energy Resources Institute, 6th km Charilaou-Thermi Road, Thessaloniki 57001, Greece; agusev@cperi.certh.gr (A.A.G.); psarras@cperi.certh.gr (A.C.P.)
[2] Aristotle University of Thessaloniki, Department of Chemistry, University Campus, Thessaloniki 54124, Greece; ktrianta@chem.auth.gr
[3] Johnson Matthey Process Technologies, Technology Center, Bourne Blvd, Savannah, GA 31408, USA; Paul.Diddams@matthey.com
* Correspondence: angel@cperi.certh.gr; Tel.: +30-2310-498305

Supplementary Material

Determination of relative crystallinity of ZSM-5 zeolites

The determination of relative crystallinity of the various steamed ZSM-5 zeolite samples was performed according to the standard ASTM D5758 method, which is based on the comparison of the sum of integrated intensities of the three dominant peaks between 23.1° and 24.3° 2θ. The following equation was used:

$$\%\text{XRD relative crystallinity of steamed ZSM} - 5 = \frac{S_x}{S_r} \times 100 \qquad (1)$$

$$S_x = integrated\ peaks\ area\ for\ the\ steamed\ ZSM - 5$$

$$S_r = integrated\ peaks\ area\ for\ the\ reference\ ZSM - 5$$

The denoted "reference ZSM-5" was in each case the parent-calcined material. In the case of steamed P/H-ZSM-5 zeolite it was the fresh P/H-ZSM-5 zeolite whereas in the case of the steamed P/ZSM-5 additive it was the fresh P/ZSM-5 additive.

Table S1. Relative crystallinity of the fresh and steamed P/ZSM-5 zeolites and additives.

Sample	Relative crystallinity, %
**P/H-ZSM-5 zeolite**	**100**
**Steamed P/H-ZSM-5 zeolite**	**86**
**Fresh P/ZSM-5 additive**	**100**
**Steamed P/ZSM-5 additive**	**95**

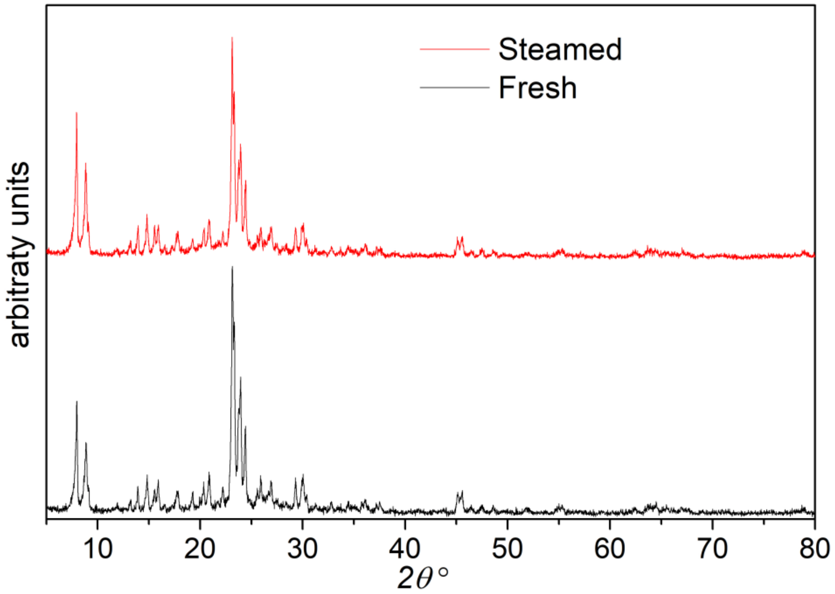

Figure S1. XRD patterns of fresh and steamed (at 770°C, 7 hrs, 50% steam/He) P/H-ZSM-5 zeolites

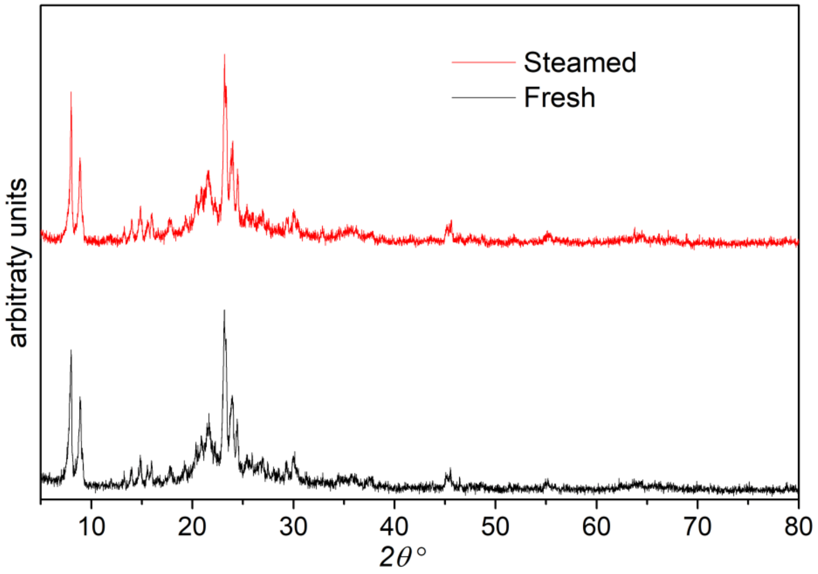

Figure S2. XRD patterns of the fresh-calcined and steamed (at 770°C, 7 hrs, 50% steam/He) P/ZSM-5 additive.

Pore size distribution curves (BJH analysis) of the catalysts

Figure S3 below show the pore size distribution curves based on the BJH analysis of the N_2 adsorption data of fresh-calcined and steamed P/H-ZSM-5 pure zeolite, of the fresh-calcined and steamed P/ZSM-5 additive and of the fresh and steamed SiO2-Al2O3; steaming conditions were 770°C, 7 hrs, 50% steam/He. The BJH curves of the fresh-calcined pure P/ZSM-5 zeolite and P/ZSM-5 additive were typical for microporous materials exhibiting no distinct peak in the micropore/small mesopore region. However, the P/ZSM-5 additive showed enhanced presence of

meso/macropores and external surface (> 100 Å). On the other hand, the steamed analogues of both the pure zeolite and the additive exhibited also a peak at about 20 Å, showing the formation of relatively smaller mesopores. With regard to the BJH curves of the SiO_2-Al_2O_3 catalyst, they exhibit a characteristic peak at about 56 Å which is shifted to about 79 Å upon steaming, due to reorganization of the mesoporous framework.

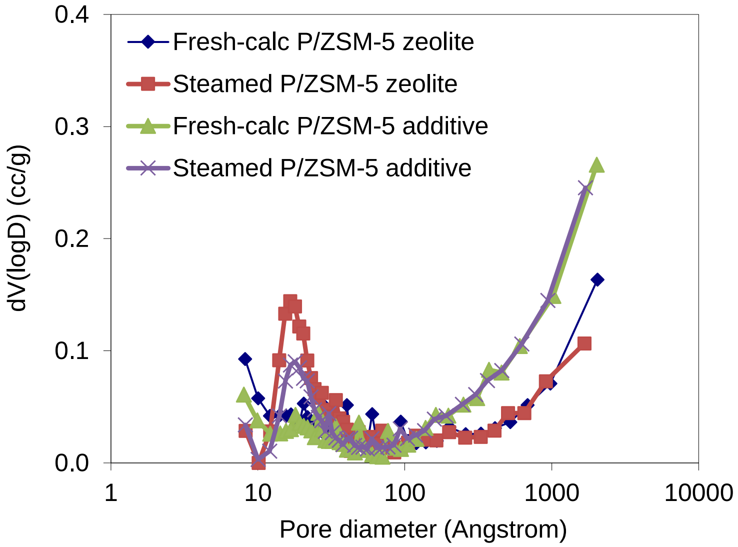

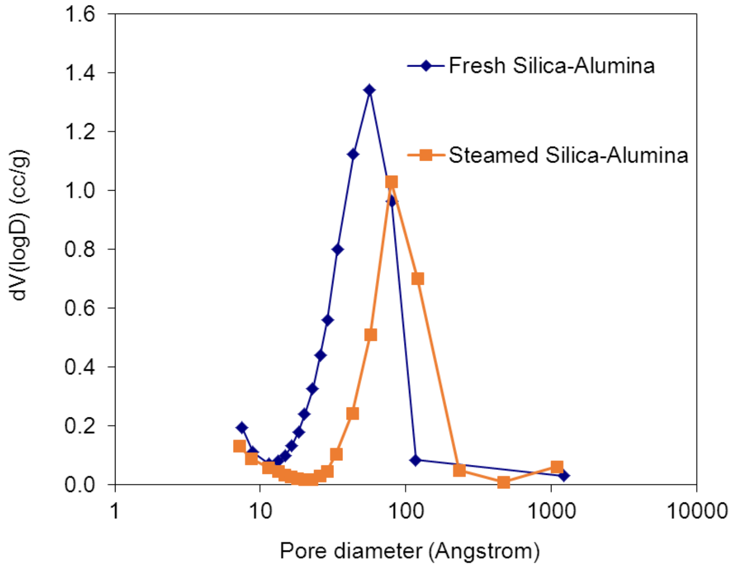

Figure S3. Pore size distribution curves based on the BJH analysis of the N_2 adsorption data of fresh-calcined and steamed P/H-ZSM-5 pure zeolite, P/ZSM-5 additive and silica-alumina; steaming conditions were 770°C, 7 hrs, 50% steam/He.
